# Process optimization and kinetics of deep fat frying conditions of sausage processed from goat meat using response surface methodology

**DOI:** 10.1002/fsn3.1145

**Published:** 2019-08-28

**Authors:** Sunday Samuel Sobowale, Tajudeen Adeniyi Olayanju, Antoine Floribert Mulaba‐Bafubiandi

**Affiliations:** ^1^ Department of Metallurgy, School of Mining, Metallurgy and Chemical Engineering, Faculty of Engineering and the Built Environment, Mineral Processing and Technology Research Centre University of Johannesburg Johannesburg South Africa; ^2^ Department of Food Technology Moshood Abiola Polytechnic Abeokuta Nigeria; ^3^ Department of Agricultural and Biosystem Engineering, College of Engineering Landmark University Omu‐Aran Nigeria

**Keywords:** deep fat frying, Goat meat, optimization, quality characteristics, sausage

## Abstract

This study investigated the effects and optimization of cooking time (30, 45, 60 min), frying temperature (150, 170, 190°C) and time (3, 6, 9 min) on the quality (moisture content, moisture loss, fat and protein content, color), textural, and sensory characteristics of deep fat fried goat meat sausage by response surface methodology (RSM) using a three‐level Box–Behnken design. The kinetic of moisture loss and fat absorption were also determined using first‐order equation. The goat meat was precooked and fried using a 2.5‐L electric deep fryer with a temperature control of ± 10°C. The results showed that all the quality, textural, and sensory characteristics of goat meat sausage investigated were significantly influenced (*p* < .05) by the frying conditions. The effective moisture diffusivity ranged from 1.22 × 10^–8^ to 2.84 × 10^–8^ m^2^/s and 2.43 × 10^–9^ to 1.22 × 10^‐8^m^2^/s for the moisture loss and fat absorption, respectively. Activation energies estimated were 71.04 to 77.76 KJ/mol and 65.82 to 67.2 KJ/mol, respectively. The frying kinetics obeyed the first‐order rate constant, and the temperature dependency of moisture loss was higher compared to fat absorption of the fried goat meat sausage in all the samples. The optimal conditions for the deep fat frying of goat meat sausage were achieved using cooking time of 45 min fat frying temperature of 150°C and time of 9 min with (*R* ^2^ > 0.9) and were the most preferred sausage sample and accepted by the sensory panelists. This study has shown that the optimal frying conditions observed could be a viable alternative for the commercialization of quality goat meat sausages and other fried meat products in the food industry.

## 1 INTRODUCTION

Deep fat frying is a unit operation which can be described as cooking of food by immersion in edible oil or fat at a higher temperature than the boiling point of water (Farkas & Hubbard, 2000). This unit operation can be regarded as a high temperature and a short time process which involves both heat and mass transfer, mainly represented by water loss and fat absorption (Vitrac, Dufour, Trystram, & Raoult‐Wack, 2002). Goats (*Capra hircus*) are known to descend from the bezoars or wild goat in the hills of western Asia (Webb, 2014). They spread widely around world making up a total of more than 850 million with about 1,156 different breeds (Devendra, 2010). As reported by Madruga and Bressan (2011), goat meat consumption globally is less than beef but serves as a staple source of red meat to humans especially in developing countries (Webb, Casey, & Simela, 2005). Meat is an excellent source of many essential nutrients, including heme iron, protein, B vitamins, and zinc, and makes an important contribution to a balanced diet (Hannah, 2012). Jihad, Ayman, and Alli (2009) reported that sausage is a prepared food usually made from ground or chopped meat's animal fat. Essien (2003) explained sausages as comminuted processed meat made of red meat or a combination of these with water, binders, and seasonings.

Although deep fat frying is unique as it confers desirable physical and sensorial qualities to food, its challenges have been attributed to the excessive retention of oil in the fried product (Akinlua, Sobowale, Adebo, & Olatidoye, 2013; Bouchon, Aguilera, & Pyle, 2003). This challenge has been economically disturbing as regards the numerous health challenges associated with the consumption of high fatty foods resulting in obesity (Hurt, Kulisek, Buchanan, & Mcclave, 2010; Swinburn, Caterson, Seidell, & James, 2004). Gadiyaram and Kannan (2004) reported that goat meat is a good source of red meat for the preparation of heart‐healthy products because of its lower fat content.

Optimization studies are enhanced by a useful technique known as response surface methodology (RSM). RSM is important in designing, formulating, developing, and analyzing new scientific studies and products which could be useful in the global food industries. However, optimization is therefore required in ensuring quick processing alongside maintaining optimum quality product (Montgomery, 2001). Quite a number of researchers (Sobukola, Awonorin, Sanni, & Bamiro, 2008; Sobowale, Adebiyi, & Adebo, 2017; Adeyanju, Olajide, & Adedeji, 2016; and Esan, Sobukola, Bakare, & Munoz, 2015) have worked extensively on optimization of deep fat fried snacks and other products. However, there has been very little or dearth of information in the literature on the use of RSM to generate mathematical models in optimizing deep fat frying conditions of sausage made from goat meat. Therefore, the aim of this study was to optimize and investigate the effect of cooking time, frying temperature and time on the quality, textural and sensory characteristics of deep fat fried sausage processed from goat meat as well as the kinetics of moisture loss and fat absorption using response surface methodology.

## 2 MATERIALS AND METHODS

### 2.1 Sample preparation

The goat meat was washed and precooked as described by Asmaa, Zzman, and Tajul (2015). The raw meat sample was divided into 200 g of three equal parts and seasoned with 2% of the whole weight before precooked. Raw meat samples are mixed according to their leanness and fat contents with the corresponding quantity of salt. About 10% of water was added. It was then mixed manually for about 3–7 min so that the salt is dissolved and the ingredients sufficiently homogenized due to continuous pushing and mixing effect. The seasoned water was allowed to boil at temperature of 100°C, and each portion was cooked differently at 30, 45, and 60 min, respectively. Each sample was packed differently labeled and was allowed to cool before grinding in a mincer. The ground samples were packaged in artificial casings and allowed to set before frying operation commences.

### 2.2 Deep fat frying operation

**Figure 1:**
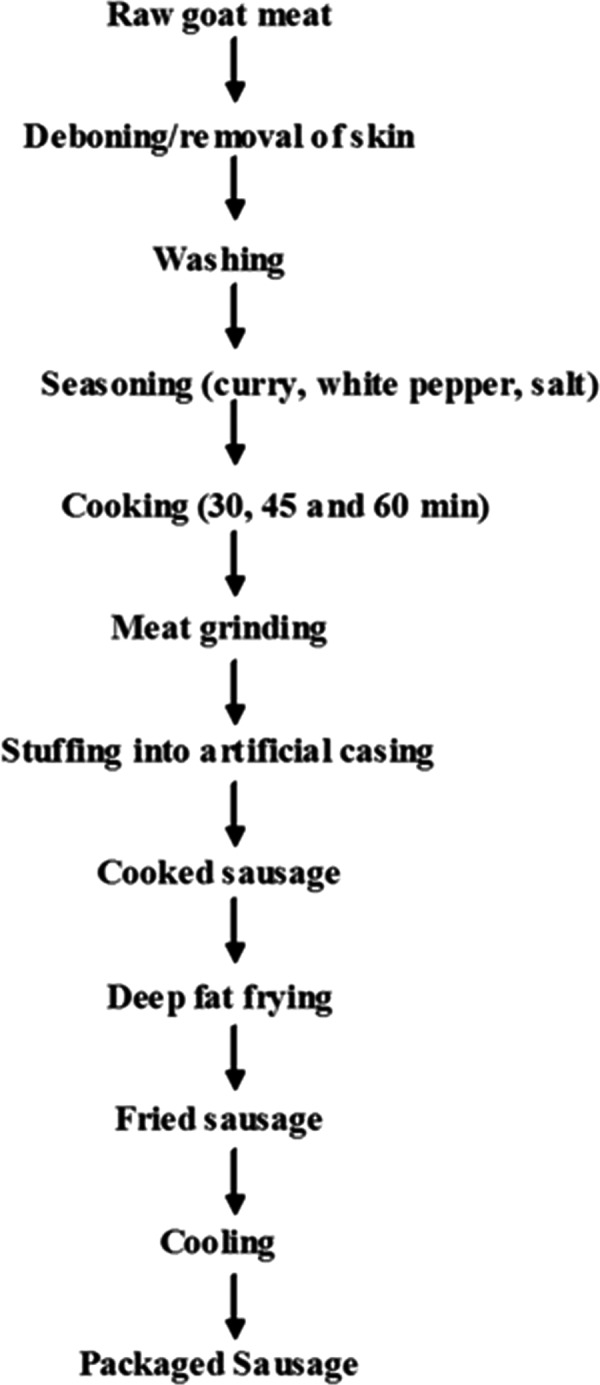
Flowchart for the processed sausage from goat meat

The deep fat frying operation was carried out using the method described by Sobowale et al. (2017). The precooked sausage samples were fried at temperatures of 150, 170, and 190°C for 3, 6, and 9 min, respectively (Table 1). Frying was done using a 2.5‐L electric deep fryer (Master Chef Mc‐df1023, Australia) with a temperature control of ± 10°C. After each frying operations, the sausage samples were drained and allowed to cool. The frying experiment was done in triplicate as shown in Figure 1.

**Table 1:** Coded values of the independent variables

Variables	Codes		
	−1	0	+1
Cooking time (min) (*X*_1_)	30	45	60
Frying temperature (^o^C) (*X*_2_)	150	170	190
Frying time (min) (*X*_3_)	3	6	9

### 2.3 Experimental design and process optimization

A Response Surface Methodology (RSM) with the applocation of Box–Behnken design was used for the design of experiment with three independent variables, including cooking time (X_1_), frying temperature (X_2_), and frying time (X_3_) using a Minitab 18, Minitab Lt, Coventry, UK, Software). The levels of each variable were established based on a series of preliminary experiments resulting in a total of 17 experimental runs (Table 2). Quadratic polynomial models were fitted to the data as necessary to obtain the regression equations. The statistical significance of the terms in the regression equations was examined by analysis of variance (ANOVA) for each response. The deep frying conditions were optimized using the numerical method of RSM based on desirability concept to obtain deep fried sausages of acceptable properties. The independent variables were kept within the experimental range while the responses were either minimized or maximized. Predictive models obtained were then used to generate the response surface plots. The mathematical model describing the relationship between the independent variables in terms of the linear, quadratic, and interaction effects is described by a second‐order polynomial equation (Anuonye, Badifu, Inyang, & Akpapunam, 2007) as presented in Equation (1).

(1) $$\begin{array}{c} Y=b_{0}+b_{1}x_{1}+b_{2}x_{2}+b_{3}x+b_{11}x_{1}^{2}+b_{22}x_{2}^{2}\\ +b_{33}x_{3}^{2}+b_{12}x_{1}x_{2}+b_{13}x_{1}x_{3}+b_{23}x_{2}x_{3}+\ldots \end{array}$$

where *Y* represents the objective response; *b*_o_, *b*_1_‐*b*_3_, *b*_11_‐*b*_33,_ and *b*_12_‐*b*_23_ are the equation regression coefficients for intercept, linear, quadratic, and interaction coefficient, respectively, and *x*_1_ – *x*_3_ are the independent variables.

**Table 2:** Coded and real values for the response surface methodology

Experimental runs	Coded values			Real values		
	*X*_1_	*X*_2_	*X*_3_	*X*_1_ (mins)	*X*_2_ (^o^C)	*X*_3_ (mins)
1	1	0	1	60	170	9
2	0	−1	1	45	150	9
3	1	0	−1	60	170	3
4	−1	1	0	30	190	6
5	0	0	0	45	170	6
6	1	−1	0	60	150	6
7	0	1	−1	45	190	3
8	0	0	0	45	170	6
9	−1	0	1	30	170	9
10	0	1	1	45	190	9
11	−1	0	−1	30	170	3
12	0	−1	−1	45	150	3
13	0	0	0	45	170	6
14	0	0	0	45	170	6
15	0	0	0	45	170	6
16	1	1	0	60	190	6
17	−1	−1	0	30	150	6

Note

*X*_1_: (cooking time); *X*_2_: (frying temperature); *X*_3_: (frying time).

### 2.4 Determination of quality characteristics

#### 2.4.1 Moisture content and moisture loss

Moisture content in the fried sausages was determined using the method described by AOAC (2004). Five grams of the sample was weighed using an electronic weighing balance (Model number: 457, Amput electronic scale) into a preweighed moisture dish and dried in an oven (NYC‐101 oven, FCD‐3000 serials, Medical and Scientific, UK) at 105°C until constant weight was achieved. The dish plus sample was removed from the oven and transferred to a desiccator to cool for 30 min. The difference between the initial and final weight of the sausage samples was recorded.

The moisture loss in the fried samples was then determined using the method of Togrul and Pehlivan (2002), while the weight of the sample before and after frying was estimated as follows:

(2) $$\%\text{Moisture loss}=\frac{M-M_{\text{e}}}{M_{\text{o}}-M_{\text{e}}}\times 100\%$$

where *M*—moisture content at frying time (*t*), *M*_e_—equilibrium moisture content (dry basis), and *M*_o_—initial moisture content.

#### 2.4.2 Fat content

The fat content of fried samples was determined on dry basis using Soxhlet fat extractor (Ankom HCl Hydrolysis System, Macedon NY, USA) (AOAC, 2000). Fried samples were ground using locally fabricated grinder. Five grams of the samples was weighed into thimbles for fat extraction in a solvent extractor using petroleum ether. Fat content was determined as ratio of the mass of extracted fat and dry matter of the sample.

#### 2.4.3 Protein content

The protein content of the fried samples was determined by standard NIP 1612 (2002) using Kjeldahl sampler system K370 and Digest system/K437, Flawil, Switzerland) as described by AOAC (2000) and reported on dry basis.

#### 2.4.4 Color measurement

Color parameters lightness (L*), redness (a*), and yellowness (b*) were measured using a colorimeter (Color Tec‐PCM, Hunterdon, NJ) as described by Krokida, Oreopoulou, Maroulis, and Marinos‐Kouris (2001a). The instrument was standardized, and the samples were placed in the sample holder. Samples were scanned at different locations to determine (L*, a*, and b*) parameters. Color difference (ΔE) was calculated using Equations (3) to (6):

(3) $$L_{0}=\frac{L*}{255}\times 100$$

(4) $$a_{0}=a^{*}\frac{240}{255}-120$$

(5) $$b_{0}=b^{*}\frac{240}{255}-120$$

(6) $$\text{Color difference}\left(\text{Hunter}\;\Delta E\right)={\left[{\left(L_{0}-L\right)}^{2}+{\left(a_{0}-a\right)}^{2}+{\left(b_{0}-b\right)}^{2}\right]}^{1/2}$$

where L*, a*, and b* are estimated color parameters determined.

### 2.5 Textural properties

The textural properties for the sausage samples were determined using a universal testing machine (M500: Testometric AX) equipped with a 100 kN load cell (Sobukola et al., 2008). Fried sausages of uniform sizes were placed on a metal support with jaws at a distance of about 35 mm apart and pressed in the middle with cylindrical flat‐end plunger (70 mm diameter) and at a speed of 2.5 mm/min.

### 2.6 Kinetics changes in moisture loss and fat absorption

First‐order kinetics equation was used to model the kinetics changes in moisture loss and fat absorption of the deep fried sausages. The moisture ratio for both fat absorption and moisture loss at different temperatures was calculated, and Ln (MR) was plotted against time for each frying temperatures. The slope of the graph was used to determine the effective moisture diffusivity (*D*_eff_) as shown in Equations (7) and (8):

(7) $$D_{\mathrm{eff}}=\frac{\text{slope}\times 4L^{2}}{\pi^{2}}$$

where *L* = ½ thickness of the sample.

Ln (*D*_eff_) was plotted against the inverse of temperature in Kelvin (1/T), where the slope of the graph was equated to *L*n (D_o_) and thus the activation energy was calculated using:

(8) $$D_{\text{eff}}=D_{o}\exp^{-\text{Ea/RT}}$$

where *D*_o_ is the pre‐exponential factor, Ea is the activation energy (KJ/mol), R is the universal gas constant (8.3143 J mol^‐1^ K^‐1^), and T is temperature in Kelvin.

The rate constants were also calculated using the Arrhenius equation:

(9) $$\text{Ln}\;k=-\left[\;\text{Ea/RT}\right]\left[1/T\right]+\text{Ln}\;k_{\text{o}}$$

Decimal reduction time (*D*) was also used to explain the kinetics changes in quality attributes. This is the time required for a decimal change in the property (quality) value at a constant temperature (Sobukola & Bouchon, 2014) and is given by:

(10) $$D=\frac{2.303}{k}$$

where *k* is the first‐order rate constant (/min), and *ko* is frequency factor. Also, the *z* value, which has been widely used in microbial kinetics, can also be used to characterize activation energies. It is defined as the temperature range in which *D*‐value changes 10‐fold (Sobukola & Bouchon, 2014) and is described as:

(11) $$Z=\frac{\left(T_{2}-T_{1}\right)}{\text{Log}(D_{1}/D_{2})}$$

where *D*_1_ and *D*_2_ are decimal reduction times at temperatures *T*_1_ and *T*_2,_ respectively.

### 2.7 Sensory evaluation of fried goat meat sausage

Prior to the sensory evaluation test, ethical clearance was obtained and informed consent of the sensory panelists was sort and obtained. Fifty panelists were asked to rank each of the samples. A 9‐point Hedonic scale for food preference (Sobowale et al., 2017; Stone & Sidel, 2004) was used to evaluate the fried sausages in terms of aroma, color, taste, crispiness, and overall acceptability. Each panelist was requested to assess each coded sample and to record the degree of differences.

### 2.8 Statistical analysis

The data obtained were subjected to analysis of variance (ANOVA) using SPSS 22 software (IBM) and analyzed in triplicate. Significant *F* tests at *p* < .05 levels of probability are determined. Minitab 18 Statistical Software (Minitab Lt.) was used in generating statistical models and also to execute ANOVA on the models at 5% confidence level. To validate the model equations obtained, the average absolute deviation (AAD), bias factor (*B*_f_), and accuracy factor (*A*_f_) were estimated as presented in Equations ((12), (13), (14)). The coefficient of fits (*R* ^2^) was also generated to compare the actual and predicted values of the models.

(12) $$\text{AAD}=\frac{\left[\sum_{i=1}^{N}\left(\frac{\left|Y_{i,\exp}-Y_{i,\text{cal}}\right|}{Y_{i,\exp}}\right)\right]}{N}$$

(13) $$B_{\text{f}}=10^{\frac{1}{N}}\sum_{i=1}^{N}\log\left(\frac{Y_{i,\text{cal}}}{Y_{i,\exp}}\right)$$

(14) $$A_{\text{f}}=10^{\frac{1}{N}}\sum_{i=1}^{N}\left|\log\left(\frac{Y_{i,\text{cal}}}{Y_{i,\exp}}\right)\right|$$

## 3 RESULTS AND DISCUSSION

### 3.1 Quality characteristics

The quality characteristics of deep fat fried sausage processed from goat meat are presented in Table 3. The moisture content of the fried sausages decreased as the frying temperature and time increased. At higher frying temperature–time, there was significant reduction in moisture content. The percentage moisture contents obtained were similar to the findings of Esan et al. (2015) in which it was observed that the fried samples of yellow fleshed cassava roots slices decreased in moisture as the frying temperature and time increased. The moisture content of a food sample is a function of its shelf stability; hence, the higher the moisture content, the faster the rate of degradation or spoilage. The moisture loss differed significantly (*p* < .05) in the fried sausage samples. This was observed to be influenced by frying conditions. The water loss was attributed to discharge of more water from the goat meat during frying process (Kim et al., 2013; Krokida et al., 2001a). In addition, more oxymyoglobin or myoglobin pigments underwent oxidation during frying and led to darker coloration. This observation is in agreement with the works of Hongbin, Da‐Wen, Ji, and Jun‐Hu (2015); Asmaa et al. (2015); and Sharma, Mulvaney, and Rizvi (2000).

**Table 3:** Experimental and predicted values of moisture content, moisture loss, fat and protein content, and color attributes of deep fat fried goat meat sausage at different frying conditions

Variables			MC(%)		ML(%)		FC(%)		PC(%)		Lightness		Redness		Yellowness		HA		CD	
X_1_ (mins)	*X*_2_ (^o^C)	*X*_3_ (mins)	Exp	Pred	Exp	Pred	Exp	Pred	Exp	Pred	Exp	Pred	Exp	Pred	Exp	Pred	Exp	Pred	Exp	Pred
60	170	9	29.59^f^ (0.33)	26.03	43.20^l^ (0.04)	43.99	33.11^d^ (0.04)	33.92	41.78^b^ (0.04)	36.68	26.35^a^ (0.37)	25.45	10.56^h^ (0.92)	11.46	12.21^ab^ (0.51)	12.12	25	27.5	171.38	167.52
45	150	9	20.23^i^ (0.21)	21.53	54.10^d^ (0.03)	54.90	33.68^c^ (0.04)	33.91	34.09^e^ (0.04)	34.59	20.38^ef^ (1.13)	20.85	10.53^h^ (0.34)	11.84	10.01^def^ (0.96)	10.64	20	25	171.02	173.19
60	170	3	50.40^b^ (0.02)	51.53	42.15^m^ (0.21)	45.88	19.38^n^ (0.01)	20.16	21.46^l^ (0.03)	23.61	18.85^f^ (0.60)	18.13	10.88^gh^ (0.04)	11.39	9.91^ef^ (0.25)	9.61	29	24.5	170.97	171.16
30	190	6	29.01^f^ (0.90)	26.76	52.14^e^ (0.02)	53.72	32.33^f^ (0.21)	33.37	33.98^e^ (0.05)	29.38	18.94^f^ (1.31)	18.50	8.99^i^ (0.73)	11.20	8.47^g^ (0.36)	9.01	26	21.5	170.84	169.11
45	170	6	28.18^f^ (0.23)	28.10	51.20^h^ (0.04)	53.05	32.82^e^ (0.03)	31.76	14.84^p^ (0.04)	30.24	15.68^g^ (0.43)	21.16	12.82^cde^ (0.07)	12.47	8.73^g^ (0.14)	10.63	20	26.4	170.88	171.19
60	150	6	42.35^c^ (0.01)	44.60	44.53^k^ (0.03)	42.95	28.25^j^ (0.04)	27.21	19.40^n^ (0.02)	24.00	20.02^ef^ (0.98)	20.45	13.40^bcd^ (0.80)	11.19	10.80^cde^ (0.45)	10.26	22	22.5	171.16	172.94
45	190	3	40.96^cd^ (0.51)	39.66	46.91^j^ (0.03)	46.11	25.21^l^ (0.02)	24.98	26.33^j^ (0.11)	25.83	20.63^ef^ (1.14)	20.16	13.40^bcd^ (0.14)	12.09	11.36^bc^ (0.34)	10.73	26	24	171.21	169.12
45	170	6	24.70^g^ (0.22)	28.10	51.86^g^ (0.01)	53.05	33.74^c^ (0.23)	31.76	42.34^a^ (0.04)	30.24	19.09^f^ (0.32)	21.16	11.94^def^ (0.04)	12.47	9.60^f^ (0.19)	10.63	24	26.4	171.02	171.19
30	170	9	16.68^j^ (0.19)	15.55	69.18^a^ (0.04)	65.45	36.02^a^ (0.03)	35.24	39.89^c^ (0.02)	37.74	16.15^g^ (1.51)	16.87	11.65^fgh^ (0.27)	11.14	7.97^g^ (0.63)	8.27	21	24.5	170.82	171.4
45	190	9	10.75^k^ (1.70)	14.13	36.33° (0.33)	38.48	35.54^b^ (0.04)	35.28	29.33^g^ (0.16)	36.08	26.65^a^ (0.99)	26.36	13.80^bc^ (0.50)	12.10	12.72^a^ (0.40)	11.88	26	24	171.31	172.72
30	170	3	40.45^d^ (0.03)	44.01	56.95^c^ (0.01)	56.16	25.79^k^ (0.01)	24.98	18.84° (0.04)	23.94	20.45^ef^ (0.73)	21.35	14.48^ab^ (0.07)	13.58	11.03^c^ (0.40)	11.12	33	25.5	171.24	159.92
45	150	3	53.33^a^ (1.61)	49.95	42.03^m^ (0.04)	39.88	19.91^m^ (0.01)	20.17	24.72^k^ (0.04)	17.97	23.93^bc^ (0.78)	24.22	12.51^def^ (0.08)	14.21	11.31^bc^ (0.25)	12.15	27	23	156.23	168.95
45	170	6	31.73^e^ (0.74)	28.10	60.31^b^ (0.04)	53.05	30.76^g^ (0.01)	31.76	26.78^i^ (0.12)	30.24	24.12^b^ (0.29)	21.16	13.47^bcd^ (1.34)	12.47	12.17^ab^ (0.76)	10.63	29	26.4	171.42	171.19
45	170	6	33.20^e^ (1.22)	28.10	52.08^ef^ (0.02)	53.05	29.22^i^ (0.01)	31.76	30.12^f^ (0.10)	30.24	22.39^cd^ (1.80)	21.16	12.90^cde^ (0.71)	12.47	11.51^bc^ (1.10)	10.63	23	26.4	171.28	171.19
45	170	6	22.67^h^ (0.02)	28.10	49.78^i^ (0.04)	53.05	32.28^f^ (0.01)	31.76	37.10^d^ (0.04)	30.24	24.52^b^ (0.36)	21.16	11.21^gh^ (0.36)	12.47	11.15^c^ (0.33)	10.63	25	26.4	171.30	171.19
60	190	6	32.40^e^ (0.34)	32.58	36.92^n^ (0.04)	33.98	30.82^g^ (0.01)	30.27	28.05^h^ (0.07)	26.40	20.87^de^ (0.67)	22.06	11.81^efg^ (0.07)	12.61	10.10^def^ (0.13)	11.03	24	28.5	171.11	173.03
30	150	6	32.60^e^ (0.29)	32.42	52.00^f^ (0.01)	54.94	29.71^h^ (0.12)	30.26	20.78^m^ (0.04)	22.43	19.86^ef^ (0.75)	18.67	15.29^a^ (1.16)	14.49	10.88^cd^ (0.32)	9.95	30	27.5	171.21	169.50

Note

Moisture content (MC), moisture loss (ML), fat content (FC), protein content (PC), hue angle (HA), color difference (CD); experimental value (Exp), predicted value (pred). Values in parentheses represent the standard deviation of duplicate measurements. Means with no common letters within a column significantly differ (*p* < .05). *X*_1:_ (cooking time); *X*_2:_ (frying temperature); and *X*_3:_ (frying time).

The fat absorption was increased while there was significant reduction in the moisture content. According to Anandh et. al. (2008), the frying temperature and time slightly affected the composition of sausage from goat meat with goat tripe and consequently increased its fat content. However, the fat content of all the fried samples studied differed significantly. These results were quite similar to that obtained and reported by Yagua and Moreira (2011) and Adeyanju et al. (2016) during deep fat frying of potato and plantain chips, respectively. The protein content decreased with the increases in the temperature and time and differed significantly (*p* < .05) with all the samples studied. These results showed similar trend with the work of Zhang, Wang, Wang, and Zhang (2014) who confirmed that frying time had considerable decrease in the protein content of fried rabbit meat.

### 3.2 Color, hue angle, and color difference

From aesthetic point of view, color is one germane quality attribute that has great influence on the acceptability of fried food by consumers. Garayo and Moreira (2002); Manzocco, Calligaris, Masrrocola, Nicolli, and Lerici (2001) reported that color is an indicative parameter used in quality control of a fried food which is affected by the reaction temperature that influence the values of L*, a*, and b*. Acceptability of the sausage meat is mostly judged by the color, but it was observed that the color of the goat meat sausage in this study (Table 3) was lower compared to the results obtained by Sobowale et al. (2017) in fried bonga fish. The effect of the frying conditions was not significant on the L* and a* values as the results do not follow a specific trend. However, there was significant reduction in the b* of the fried sausage samples at temperature of 150°C as frying time increased. The L*, a*, b* values increased progressively with increasing frying temperature and time. However, at lower frying temperature, a* value decreased with increasing frying time and cooking time. The hue angle was smaller compared to the work of Manjuanatha, Ravi, Negi, Raju, and Bawa (2012) who investigate the kinetics of moisture loss and oil uptake in Gethi strips. It was observed that the hue angle decreased with increase in frying temperature and cooking time. The color difference was influenced significantly (*p* < .05) with increase in frying temperature and time as it increased linearly and the color difference was greater compared to the work of Adeyanju et al. (2016). Baik and Mittal (2003) also stated that increase in the color difference could be attributed to the high temperature and low moisture content observed in the sample.

### 3.3 Textural properties

The textural properties (hardness or force @peak, deflection @peak, energy to peak, adhesiveness, chewiness, cohesiveness, fracturability, gumminess, springiness, stringiness, force @break, and energy to break) of the fried goat meat sausages are presented in Tables 4 and 5. Texture is an important and desirable attribute of food products (Sobowale et al., 2018). Processing conditions and ingredient formulations have a direct impact on the textural behavior of food products (Pandey, Harilali, & Radhakrishna, 2014). Texture is also important in sensorial quality analysis which gives insight into the perception of consumers especially when fried foods are considered (Krokida, Oreopoulou, & Maroulis, 2000b). When the sausage samples were subjected to frying process, leakage of cellular organelles as well as expulsion of entrapped air took place, as a result drip was released from the fried sausage, and consequently, the texture changed and the sausage became darker. Among all textural properties, hardness regarded as the most important attribute of meat products (Hasan, Lutfiye, Ismet, Faith, and Safa, 2017). In this study, the hardness of the fried sausage samples was greatly influenced by the cooking and frying time. A lower cooking and frying time brought about a decrease in the hardness of the fried sample. There was no significant difference (*p* > .05) in the deflection @peak as well as the energy to peak of the fried goat meat sausage samples. The energy to peak was also observed to be increasing at higher frying time. There was no significant difference (*p* < .05) in the adhesiveness of the fried sausage samples. Kushmi, Pawar, and Modi (2013) found a much lower adhesiveness in conventionally fried chicken breast and leg meat with no significant difference among the samples. Chewiness of the fried goat meat sausages differed significantly (*p* < .05) and which was similar to the findings of Foegeding et al. (2011). Cohesive and springiness behavior reflects the viscoelastic properties of the food products (Pandey et al., 2014). Low values of cohesiveness of the fried samples were observed, and this was slightly similar to the results of conventionally fried chicken breast and leg meat as reported by Kushmi et al. (2013). There was significant difference (*p* < .05) in the fracturability of the fried sausage samples as the frying temperature and time increase. Gumminess is the product of hardness and cohesiveness, and it enhances the energy needed to break the semisolid food before swallowing (Hasan et al., 2017). Gumminess values varied significantly (*p* < .05) among the samples. Springiness of the samples showed a significant difference (*p* < .05). The springiness decreased as the frying temperature and time increase. The force @break of the fried sausage samples exhibited no significant difference but, increased initially followed by a decrease, the frying temperature and time were increasing. In the case of energy to break, a considerable increase was observed in all the fried samples.

**Table 4:** Experimental values of the textural properties for deep fat fried sausage at different frying conditions

Runs	*X*_1_ (mins)	*X*_2_ (^o^C)	*X*_3_ (mins)	Hardness (*N*)	Deflection @peak (mm)	Energy to peak (Nm)	Adhesiveness (*N*/s)	Chewiness (*N*)	Cohesiveness	Fracturability (*N*)	Gumminess (*N*)	Springiness	Stringiness (mm)	Force @break (*N*)	Energy to break (Nm)
1	60	170	9	28.46^a^ (7.3)	2.58^b^ (0.46)	0.04^ab^ (0.00)	26.04^a^ (25.48)	0.37^f^ (0.32)	0.17^e^ (0.05)	14.27^a‐d^ (0.08)	4.96^cd^ (2.70)	0.07^d^ (0.04)	4.75^a^ (0.1)	3.10^b^ (3.14)	0.08^a^ (0.02)
2	45	150	9	3.22^c^ (0.80)	4.73^a^ (0.04)	0.01^b^ (0.00)	1.38^b^ (0.20)	1.71^ef^ (0.70)	0.63^abc^ (0.02)	3.22^e^ (0.80)	2.02^d^ (0.57)	0.83^ab^ (0.11)	3.67^cde^ (0.1)	0.19^a^ (0.01)	0.01^d^ (0.00)
3	60	170	3	3.99^c^ (0.02)	4.75^a^ (0.00)	0.01^b^ (0.00)	1.99^b^ (0.4)	2.29^de^ (0.24)	0.72^ab^ (0.04)	3.58^e^ (0.55)	2.86^d^ (0.13)	0.80^ab^ (0.04)	3.42^c‐f^ (0.33)	0.26^a^ (0.08)	0.01^d^ (0.00)
4	30	190	6	16.79^abc^ (0.46)	4.72^a^ (0.05)	0.06^a^ (0.01)	3.42^b^ (2.62)	5.81^ab^ (1.53)	0.54^cd^ (0.07)	15.52^ab^ (1.26)	9.10^ab^ (0.81)	0.63^bc^ (0.11)	3.51^c‐f^ (0.59)	0.44^a^ (0.34)	0.07^abc^ (0.01)
5	45	170	6	8.34^bc^ (2.21)	4.75^a^ (0.05)	0.03^b^ (0.01)	3.01^b^ (0.97)	3.20^cde^ (0.00)	0.56^cd^ (0.13)	7.06^b‐e^ (1.95)	4.49^cd^ (0.08)	0.73^ab^ (0.01)	3.81^bc^ (0.17)	0.42^a^ (0.18)	0.03^d^ (0.01)
6	60	150	6	5.28^c^ (0.58)	4.75^a^ (0.04)	0.01^b^ (0.00)	1.59^b^ (0.13)	2.99^cde^ (0.42)	0.64^abc^ (0.01)	4.89^de^ (1.13)	3.35^d^ (0.43)	0.89^a^ (0.01)	3.30^c‐f^ (0.17)	0.19^a^ (0.02)	0.01^d^ (0.00)
7	45	190	3	6.02^bc^ (0.66)	4.75^a^ (0.04)	0.02^b^ (0.01)	2.37^b^ (1.17)	3.83^cd^ (0.06)	0.74^a^ (0.02)	4.69^de^ (0.42)	4.39^cd^ (0.35)	0.88^a^ (0.06)	3.11^ef^ (0.17)	0.36^a^ (0.28)	0.02^d^ (0.01)
8	45	170	6	12.17^bc^ (5.44)	4.77^a^ (0.04)	0.04^ab^ (0.02)	2.73^b^ (2.31)	5.76^ab^ (0.49)	0.65^abc^ (0.14)	9.56^b‐e^ (5.15)	7.57^abc^ (1.82)	0.79^ab^ (0.25)	3.77^bcd^ (0.43)	0.37^a^ (0.28)	0.04^cd^ (0.02)
9	30	170	9	5.90^bc^ (1.07)	4.77^a^ (0.01)	0.02^b^ (0.00)	2.08^b^ (0.51)	3.12^cde^ (0.49)	0.69^abc^ (0.01)	5.44^cde^ (1.36)	4.02^cd^ (0.76)	0.78^ab^ (0.02)	3.16^def^ (0.24)	0.27^a^ (0.91)	0.02^d^ (0.00)
10	45	190	9	27.19^a^ (18.43)	2.42^b^ (1.63)	0.04^ab^ (0.04)	4.48^b^ (2.31)	0.33^f^ (0.08)	0.22^e^ (0.04)	20.87^a^ (12.40)	5.55^bcd^ (2.76)	0.07^d^ (0.02)	1.72^a^ (0.00)	0.56^a^ (0.28)	0.07^ab^ (0.04)
11	30	170	3	2.98^c^ (0.28)	4.71^a^ (0.06)	0.01^b^ (0.00)	1.47^b^ (0.01)	1.89^ef^ (0.19)	0.71^ab^ (0.01)	2.66^e^ (0.18)	2.12^d^ (0.24)	0.89^a^ (0.01)	3.16^def^ (0.17)	0.20^a^ (0.00)	0.01^d^ (0.00)
12	45	150	3	7.53^bc^ (1.35)	4.77^a^ (0.06)	0.02^b^ (0.00)	1.76^b^ (0.30)	3.87^cd^ (0.59)	0.66^abc^ (0.03)	6.84^b‐e^ (2.33)	4.97^cd^ (0.69)	0.78^ab^ (0.01)	3.49^c‐f^ (0.1)	0.23^a^ (0.04)	0.02^d^ (0.00)
13	45	170	6	15.83^abc^ (1.26)	4.76^a^ (0.00)	0.04^ab^ (0.01)	1.72^b^ (0.17)	6.75^a^ (0.14)	0.62^abc^ (0.04)	15.83^ab^ (1.26)	9.79^a^ (0.27)	0.69^ab^ (0.03)	3.72^b‐e^ (0.04)	0.22^a^ (0.02)	0.04^cd^ (0.01)
14	45	170	6	15.92^abc^ (5.52)	4.76^a^ (0.00)	0.04^ab^ (0.01)	2.28^b^ (1.64)	6.70^a^ (0.07)	0.64^abc^ (0.09)	15.92^ab^ (5.52)	9.84^a^ (1.96)	0.70^ab^ (0.13)	3.75^bcd^ (0.53)	0.28^a^ (0.2)	0.04^cd^ (0.01)
15	45	170	6	15.02^abc^ (5.11)	4.73^a^ (0.03)	0.04^ab^ (0.02)	4.35^b^ (0.85)	5.91^ab^ (1.87)	0.58^bcd^ (0.00)	15.02^abc^ (5.11)	8.73^ab^ (2.99)	0.68^ab^ (0.04)	3.87^bc^ (0.04)	0.55^a^ (0.11)	0.04^bcd^ (0.01)
16	60	190	6	19.13^ab^ (6.99)	3.76^a^ (1.35)	0.04^ab^ (0.01)	5.43^b^ (6.22)	4.28^bc^ (1.63)	0.48^d^ (0.02)	7.13^b‐e^ (4.71)	8.99^ab^ (2.94)	0.47^c^ (0.03)	4.33^ab^ (0.16)	0.68^a^ (0.8)	0.06^abc^ (0.01)
17	30	150	6	4.70^c^ (1.95)	4.74^a^ (0.01)	0.01^b^ (0.00)	2.18^b^ (1.00)	2.71^cde^ (0.95)	0.68^abc^ (0.00)	4.11^e^ (1.12)	3.18^d^ (1.30)	0.86^a^ (0.05)	2.99^f^ (0.00)	0.33^a^ (0.13)	0.02^d^ (0.01)

Note

Values are means of duplicate determinations ± *SD*; Means value with different superscript within the same column are significantly different at *p* ≤ .05; *X*_1_: (cooking time); *X*_2_: (frying temperature); and *X*_3_: (frying time).

**Table 5:** Predicted values of the textural properties for deep fat fried sausage at different frying conditions

Runs	*X*_1_ (mins)	*X*_2_ (^o^C)	*X*_3_ (mins)	Hardness (*N*)	Deflection @peak (mm)	Energy to peak (Nm)	Adhesiveness (*N*/s)	Chewiness (*N*)	Cohesiveness	Fracturability (*N*)	Gumminess (*N*)	Springiness	Stringiness (mm)	Force @break (*N*)	Energy to break (Nm)
1	60	170	9	24.56	2.70	0.30	20.29	0.11	0.22	11.99	4.03	0.16	4.72	2.41	0.01
2	45	150	9	4.10	4.59	0.27	4.01	1.61	0.62	3.57	1.67	0.82	3.51	0.52	0.06
3	60	170	3	2.71	4.93	0.28	1.972	3.05	0.76	1.53	3.38	0.88	3.27	0.26	0.06
4	30	190	6	13.77	4.69	0.38	0.36	5.21	0.58	13.58	7.82	0.71	3.31	0.08	0.47
5	45	170	6	13.46	4.75	0.3	2.82	5.66	0.61	12.68	8.08	0.71	3.78	0.37	0.04
6	60	150	6	8.30	4.76	0.29	4.64	3.57	0.59	6.83	4.63	0.81	3.50	0.55	0.16
7	45	190	3	5.14	4.87	0.29	0.33	3.93	0.74	4.35	4.74	0.89	3.29	0.03	0.09
8	45	170	6	13.46	4.75	0.3	2.82	5.66	0.61	12.68	8.08	0.71	3.78	0.37	0.04
9	30	170	9	7.16	4.59	0.29	2.10	2.36	0.64	7.50	3.45	0.70	3.31	0.28	0.09
10	45	190	9	28.94	2.61	0.32	7.52	1.69	0.22	20.75	7.35	0.05	4.77	0.92	0.15
11	30	170	3	6.88	4.59	0.29	7.21	2.39	0.66	4.95	3.05	0.79	3.19	0.89	0.09
12	45	150	3	5.78	4.56	0.28	1.28	2.52	0.66	6.96	3.17	0.79	3.44	0.13	0.06
13	45	170	6	13.46	4.75	0.3	2.82	5.66	0.61	12.68	8.08	0.71	3.78	0.36	0.04
14	45	170	6	13.46	4.75	0.3	2.82	5.66	0.61	12.68	8.08	0.71	3.78	0.37	0.04
15	45	170	6	13.46	4.75	0.3	2.82	5.66	0.61	12.68	8.08	0.71	3.78	0.37	0.04
16	60	190	6	21.28	3.45	0.31	8.13	3.42	0.42	9.53	8.17	0.38	4.31	1.02	0.06
17	30	150	6	2.55	5.0538	0.28	0.54	3.57	0.74	1.71	4.05	0.95	3.01	0.01	0.01

Note

Values are means of duplicate determinations ± *SD*; Means value with different superscript within the same column are significantly different at *p* ≤ .05; *X*_1_: (cooking time); *X*_2_: (frying temperature); and *X*_3_: (frying time).

### 3.4 Kinetics of quality changes

The kinetics of moisture loss and fat absorption of deep fat fried goat meat sausages at different frying conditions are presented in Table 6 and 7. The effective moisture diffusivity (*D*_eff_) for moisture loss and fat absorption at temperatures 150, 170, and 190°C were 2.84 × 10^–8^ m^2^/s, 1.22 × 10^–8^ m^2^/s, and 2.84 × 10^–8^ m^2^/s and 2.43 × 10^–9^ m^2^/s, 8.10 × 10^–9^ m^2^/s, and 1.22 × 10^–8 ^m^2^/s, respectively. The activation energies ranged from 71.04 to 77.76 KJ/mol and from 65.82 to 67.20 KJ/mol, respectively, with the rate constants of 0.0592 to 0.0596/s and 0.447 to 0.4496/s, respectively. The decimal reduction times (*D* value) and thermal resistance constant (*Z*‐value) for both moisture loss and fat absorption ranged from 38.64 to 38.90 s and 842.1 to 875.0 K and 5.13 to 5.15 s and 800.0 to 842.0 K, respectively.

**Table 6:** Kinetic of moisture loss during the deep fat frying of goat meat sausage

Temperature (^o^C)	*D*_eff_ (*m* ^2^/s)	Ea (KJ/mol)	*K* (/s)	*D* value (s)	*Z* value (K)
150	2.84 × 10^–8^	71.04	0.0596	38.64	857.14
170	1.22 × 10^–8^	77.67	0.0595	38.71	875
190	2.84 × 10^–8^	77.76	0.0592	38.90	842.1

Note

*D_eff_* (effective moisture diffusivity), Ea (activation energy), *K* (rate constant), *D* value (decimal reduction time), and *Z* value (thermal resistance constant).

**Table 7:** Kinetic of fat absorption during the deep fat frying of goat meat sausage

Temperature (^o^C)	*D*_eff_ (m^2^/s)	Ea (KJ/mol)	*K* (/s)	*D* value (s)	*Z* value (K)
150	2.43 × 10^–9^	67.17	0.447	5.15	800
170	8.10 × 10^–9^	65.82	0.448	5.14	823
190	1.22 × 10^–8^	67.20	0.449	5.13	842

Note

*D*_eff_ (effective moisture diffusivity), Ea (activation energy), *K* (rate constant), *D* value (decimal reduction time), and *Z* value (thermal resistance constant).

The *D*_eff_ for moisture loss reduced initially and then increased with an increase in frying temperature, while that of the fat absorption progressively increased as the temperature increases. Similar observation was reported by Lopez, Iguaz, Esnoz, and Virseda (2000) during the deep fat frying of rice crackers. In this study, it was observed that deep frying of the goat meat sausage resulted in moisture transfer out of the fried product internally by diffusion and externally by evaporation in the form of vapors. This is in agreement with the report of Pathare and Sharma (2006). Since frying temperatures are typically above 100°C, it is expected that moisture would be transferred from porous solid food material as liquid and vapors. However, *D*_eff_ is known as the total diffusivity of moisture in liquid and vapors but its estimation is usually difficult as shown in this study (Thomas, Anjaneyulu, & Kondaiah, 2006). Thus, estimation of *D*_eff_ shown to be further complicated by the complexity of food–oil interaction and typically high frying oil temperatures.

Activation energy increased significantly with increase in temperature. These are in agreement with the work of Sobukola et al. (2008) on atmospherically fried yellow fleshed cassava root slices. It can also be deduced that moisture loss requires more energy than fat absorption. The lower activation energies observed indicate that frying of goat meat sausage requires less energy and the process is less sensitive and dependent on temperature changes (Vitrac et al., 2002).

Both mass transfer phenomena (moisture loss and fat absorption) that take place during frying of goat meat sausage were described by empirical first‐order rate constant. Frying temperature and time are the process variables that affect significantly the rate constant. The rate constants obtained for moisture loss show a linear decrease with increasing temperature. However, fat absorption was significantly higher (*p* < .05) than the moisture loss as it indicated an increase with increasing temperature. This result is similar to the findings of Debnath, Bhat, and Rastogi (2003) when investigating the effect of predrying on kinetics of moisture loss during deep fat frying of chickpea flour‐based snack food. The values obtained for fat absorption are higher compared to the ones reported by Debnath et al. (2003). These variations may be due to the different frying conditions, processing methods, and possibly the type of fried food product. The D values for moisture loss reveal an increase as the frying temperature increased, while the *Z* values obtained also indicate an initial increase but further reduced at an elevated frying temperature of 190°C. On the other hand, the *D* value for fat absorption decreased linearly as the frying temperature increased and *Z* values showed similar behavior as in the case of moisture loss. These values differed from those reported by Sobukola et al. (2008). The frying kinetics obeyed the first‐order rate constant, and the temperature dependency of moisture loss was higher compared to fat absorption of the fried goat meat sausage in all the samples.

#### 3.4.1 Statistical models and validation

**Figure 2:**
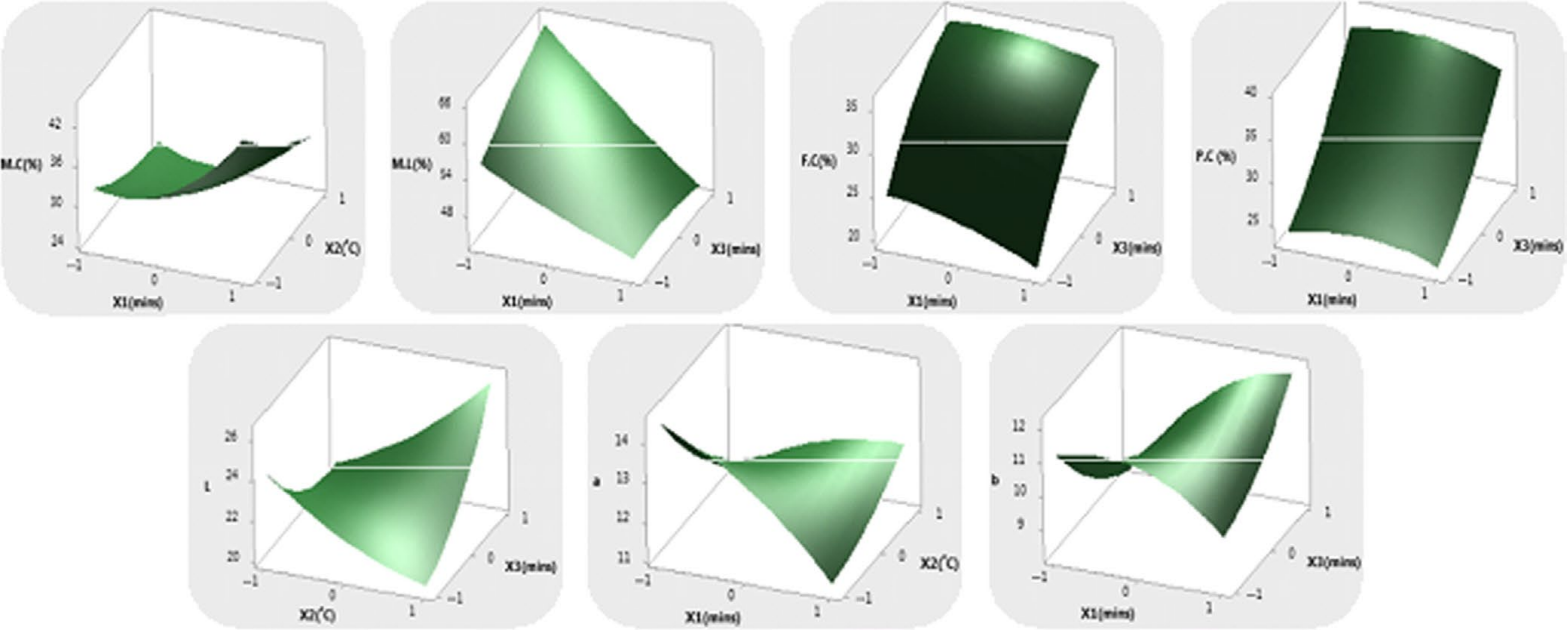
Response surface plots of MC—moisture content, ML—moisture loss, FC—fat content, PC—protein content, L*—lightness, a*—redness, b*—yellowness

**Figure 3:**
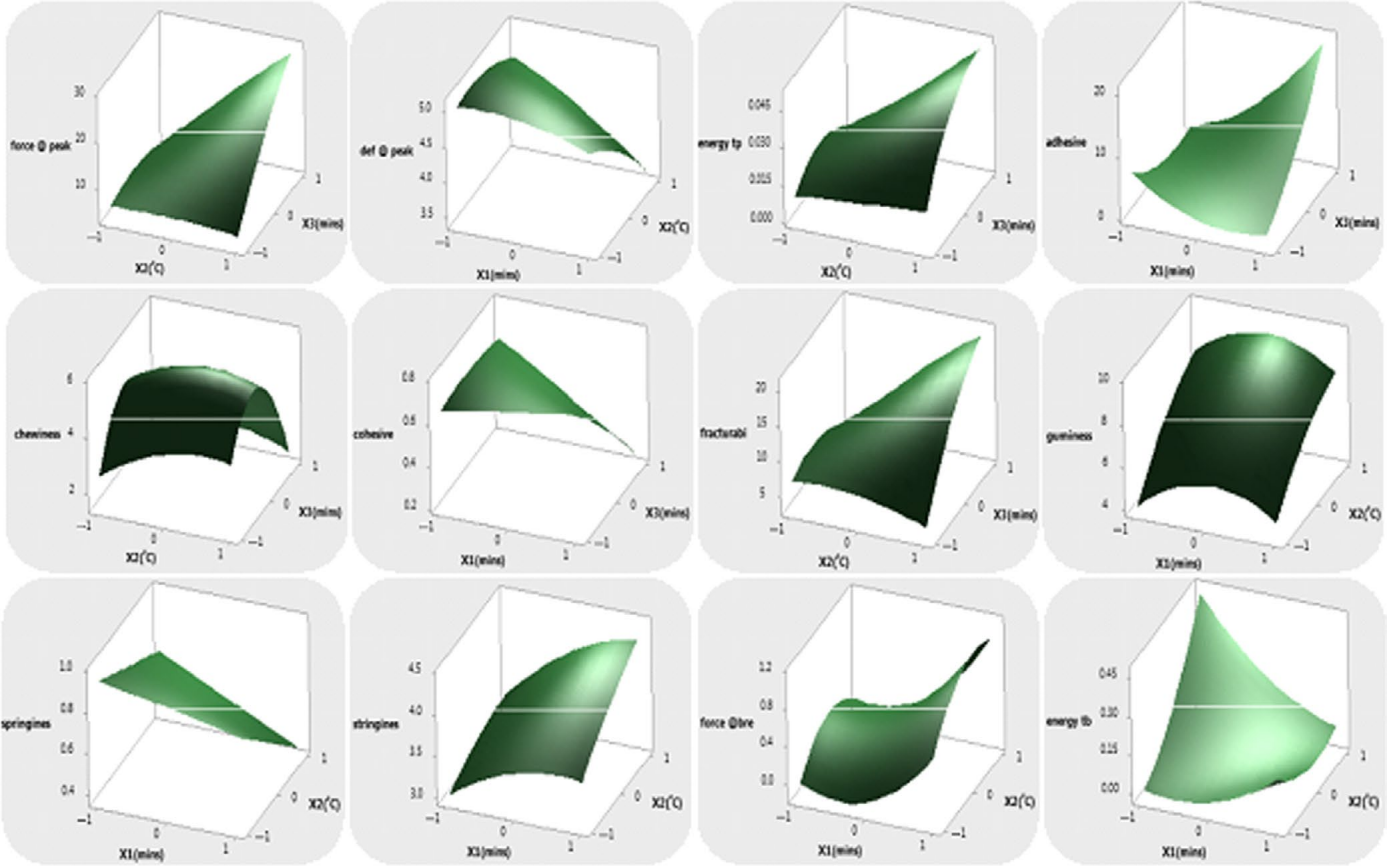
Response surface plots of textural properties (hardness or force @peak, def @peak, energy to peak, adhesiveness and chewiness, cohesiveness, fracturability, gumminess, springiness, stringiness, force @ break, energy to break

The effects of three independent extrusion variables: cooking time (*X*_1_), frying temperature (*X*_2_), and frying time (*X*_3_) on the following responses (moisture content, moisture loss, protein content, fat content, lightness, redness, yellowness, hue angle, color difference, hardness or force @peak, def @peak, energy to peak, adhesiveness, chewiness, cohesiveness, fracturability, gumminess, springiness, stringiness, force @break, energy @break) were investigated in this study. The different models representing each response are presented in Equations (15), (16), (17), (18), (19), (20), (21), (22), (23), (24), (25), (26), (27), (28), (29), (30), (31), (32), (33), (34), (35), while the response surface plots are depicted in Figures 2 and 3.

(15) $$\begin{array}{c} \text{Moisture content}=28.096+4.5X_{1}-4.4238X_{2}-13.4863X_{3}+4.4783X_{1^{2}}+1.5157X_{2^{2}}\\ +1.7057X_{3^{2}}-1.59X_{1}X_{2}+0.74X_{1}X_{3}+0.7225X_{2}X_{3} \end{array}$$

(16) $$\begin{array}{c} \text{Moisture loss}=53.046-7.9338X_{1}-2.545X_{2}+1.8463X_{3}+0.6895X_{1^{2}}-7.338X_{2^{2}}\\ -0.8655X_{3^{2}}-1.9375X_{1}X_{2}-2.795X_{1}X_{3}-5.6625X_{2}X_{3} \end{array}$$

(17) $$\begin{array}{c} \text{Protein content}=30.236-0.35X_{1}+2.3375X_{2}+6.7175X_{3}-1.4042X_{1^{2}}-3.2793X_{2^{2}}\\ +1.6608X_{3^{2}}-1.1375X_{1}X_{2}-0.1825X_{1}X_{3}-1.5925X_{2}X_{3} \end{array}$$

(18) $$\begin{array}{c} \text{Fat content}=31.764-1.5363X_{1}+1.5438X_{2}+6.0075X_{3}-0.7482X_{1^{2}}-0.7383X_{2^{2}}\\ -2.4407X_{3^{2}}-0.0125X_{1}X_{2}+0.875X_{1}X_{3}-0.86X_{2}X_{3} \end{array}$$

(19) $$\begin{array}{c} \text{Lightness}=21.16+1.3363X_{1}+0.3625X_{2}+0.7088X_{3}-1.8425X_{1^{2}}+0.6050X_{2^{2}}\\ +1.1325X_{3^{2}}+0.4425X_{1}X_{2}+2.9500X_{1}X_{3}+2.3925X_{2}X_{3} \end{array}$$

(20) $$\begin{array}{c} \text{Redness}=12.4680-0.4700X_{1}-00.4662X_{2}-0.5913X_{3}-0.3815X_{1^{2}}+0.2860X_{2^{2}}\\ -0.1940X_{3^{2}}+1.1775X_{1}X_{2}+0.6275X_{1}X_{3}+0.595X_{2}X_{3} \end{array}$$

(21) $$\begin{array}{c} \text{Yellowness}=10.6320+0.5838X_{1}-0.0438X_{2}-0.0875X_{3}-0.8198X_{1^{2}}+0.2503X_{2^{2}}\\ +0.4678X_{3^{2}}+0.4275X_{1}X_{2}+1.3400X_{1}X_{3}+0.6650X_{2}X_{3} \end{array}$$

(22) $$\begin{array}{c} \text{Hue angle}=26.40+0.50X_{1}+0.00X_{2}+0.50X_{3}+0.05X_{1^{2}}-1.45X_{2^{2}}-0.95X_{3^{2}}+3.0X_{1}X_{2}\\ +1.00X_{1}X_{3}-0.50X_{2}X_{3} \end{array}$$

(23) $$\begin{array}{c} \text{Color difference}=171.19+1.84X_{1}-0.075X_{2}+1.96X_{3}-1.77X_{1^{2}}+1.72X_{2^{2}}-1.92X_{3^{2}}\\ +0.12X_{1}X_{2}-3.78X_{1}X_{3}-0.16X_{2}X_{3} \end{array}$$

(24) $$\begin{array}{c} \text{Hardness}=13.456+3.3112X_{1}+6.05X_{2}+5.5313X_{3}-1.3218X_{1^{2}}-0.6592X_{2^{2}}-1.8067X_{3^{2}}\\ +0.44X_{1}X_{2}+5.3925X_{1}X_{3}+6.37X_{2}X_{3} \end{array}$$

(25) $$\begin{array}{c} \text{Def@peak}=4.754-0.3863X_{1}-0.4175X_{2}-0.5588X_{3}-0.112X_{1^{2}}-0.1545X_{2^{2}}-0.441X_{3^{2}}\\ -0.2375X_{1}X_{2}-0.56X_{1}X_{3}-0.5725X_{2}X_{3} \end{array}$$

(26) $$\begin{array}{c} \text{Energy to peak}=0.30+0.00X_{1}+0.0125X_{2}+0.0075X_{3}-0.00X_{1^{2}}-0.00X_{2^{2}}-0.010X_{3^{2}}\\ -0.005X_{1}X_{2}+0.005X_{1}X_{3}+0.01X_{2}X_{3} \end{array}$$

(27) $$\begin{array}{c} \text{Adhesiveness}=2.8160+3.2375X_{1}+1.0975X_{2}+3.30X_{3}+2.8657X_{1^{2}}-2.5293X_{2^{2}}+2.2108X_{3^{2}}\\ +0.6475X_{1}X_{2}+5.8575X_{1}X_{3}+0.6225X_{2}X_{3} \end{array}$$

(28) $$\begin{array}{c} \text{Chewiness}=5.6620-0.4475X_{1}+0.3725X_{2}-0.7900X_{3}-1.1197X_{1^{2}}-0.5997X_{2^{2}}-2.6248X_{3^{2}}\\ -0.4475X_{1}X_{2}-0.7875X_{1}X_{3}-0.3325X_{2}X_{3} \end{array}$$

(29) $$\begin{array}{c} \text{Cohesiveness}=0.61-0.0775X_{1}-0.08X_{2}-0.14X_{3}-0.01X_{1^{2}}-0.02X_{2^{2}}-0.03X_{3^{2}}-0.005X_{1}X_{2}\\ -0.13X_{1}X_{3}-0.120X_{2}X_{3} \end{array}$$

(30) $$\begin{array}{c} \text{Fracturability}=12.6780+0.2675X_{1}+3.6425X_{2}+3.2525X_{3}-3.5903X_{1^{2}}-1.1753X_{2^{2}}-2.6002X_{3^{2}}\\ -2.2925X_{1}X_{2}+1.9775X_{1}X_{3}+4.9475X_{2}X_{3} \end{array}$$

(31) $$\begin{array}{c} \text{Gumminess}=8.082+0.21750X_{1}+1.81375X_{2}+0.27625X_{3}-1.33475X_{1^{2}}-0.59225X_{2^{2}}\\ -3.25725X_{3^{2}}-0.07X_{1}X_{2}+0.05X_{1}X_{3}+1.0275X_{2}X_{3} \end{array}$$

(32) $$\begin{array}{c} \text{Springiness}=0.71200-0.116250X_{1}-0.16500X_{2}-0.201250X_{3}-0.00100X_{1^{2}}+0.0015X_{2^{2}}\\ -0.07600X_{3^{2}}-0.0475X_{1}X_{2}-0.155X_{1}X_{3}-0.2175X_{2}X_{3} \end{array}$$

(33) $$\begin{array}{c} \text{Stringiness}=3.7800+0.37250X_{1}+0.27875X_{2}+0.38875X_{3}-0.18750X_{1^{2}}-0.0600X_{2^{2}}\\ +0.0300X_{3^{2}}+0.12750X_{1}X_{2}+0.33250X_{1}X_{3}+0.3550X_{2}X_{3} \end{array}$$

(34) $$\begin{array}{c} \text{Force@break}=-0.36800-0.37375X_{1}-0.13750X_{2}-0.38375X_{3}-0.33225X_{1^{2}}+0.29025X_{2^{2}}\\ -0.25725X_{3^{2}}-0.09500X_{1}X_{2}-0.69250X_{1}X_{3}-0.060X_{2}X_{3} \end{array}$$

(35) $$\begin{array}{c} \text{Energy to break}=0.03600-0.0625X_{1}+0.0900X_{2}+0.0150X_{3}+0.06950X_{1^{2}}+0.06950X_{2^{2}}\\ -0.07550X_{3^{2}}-0.140X_{1}X_{2}+0.015X_{1}X_{3}+0.015X_{2}X_{3} \end{array}$$

The computed coefficients of regression (*R* ^2^) were greater than 0.9, implying a better consonance between the actual and predicted values (Tables 8 and 9). Observation drawn from the coefficient of regression model indicates positive quadratic effect on frying temperature and time, while there was no significant effect (*p* > .05) on the cooking time in all the samples studied. Previous studies have affirmed that a good fit of empirical model and experimental data are depicted by *R* ^2^ > 0.9 (Odunmbaku et al., 2018; Sobowale et al., 2017, 2018). Apart from high R^2^ values which indicated validity of the model, other measures including bias factor (*B*_f_) and accuracy factor (*A*_f_) judged by closeness to unity (1) and not excluding average absolute deviation (AAD) (values close to zero). All the results gave acceptable estimated (predicted) and actual data (experimental). The range of the values obtained further indicates the adequacy of the models for describing the investigated samples.

**Table 8:** Coefficient of regression and *R* ^2^, AAD, *B*_f,_ *A*_f_ for the mathematical models of the responses (moisture content, moisture loss, fat and protein content, and color)

Coefficient	Moisture content	Moisture loss	Fat content	Protein content	Lightness	Redness	Yellowness	Hue angle	Color difference
*b*_0_	28.0960	53.0460	31.7640	30.2360	21.1600	12.4680	10.6320	26.400	171.19
*b*_1_	4.5000	−7.9338	−1.5363	−0.3500	1.3363	−0.4700	0.5838	0.5000	1.8400
*b*_2_	−4.4238	−2.5450	1.5438	2.3375	0.3625	−0.4662	−0.0438	0.000	−0.075
*b*_3_	−13.4863	1.8463	6.0075	6.7175	0.7088	−0.5913	−0.0875	0.5000	1.9600
*b*_11_	4.4783	0.6895	−0.7482	−1.4042	−1.8425	−0.3815	−0.8198	0.0500	−1.7700
*b*_22_	1.5157	−7.3380	−0.7383	−3.2793	0.6050	0.2860	0.2503	−1.4500	1.7200
*b*_33_	1.7057	−0.8655	−2.4407	1.6608	1.1325	−0.1940	0.4678	−0.9500	−1.9200
*b*_12_	−1.5900	−1.9375	−0.0125	−1.1375	0.4425	1.1775	0.4275	3.0000	0.1200
*b*_13_	0.7400	−2.7950	0.8750	−0.1825	2.9500	0.6275	1.3400	1.0000	−3.7800
*b*_23_	0.7225	−5.6625	−0.8600	−1.5925	2.3925	0.5950	0.6650	−0.5000	−0.1600
*R* ^2^ (%)	92.94	88.31	95.22	43.00	61.10	38.36	56.04	28.11	74.00
AAD	0.09	0.15	0.03	0.19	0.07	0.09	0.07	0.14	0.02
B_f_	1.01	1.00	1.00	1.02	1.00	1.01	1.00	1.03	1.02
A_f_	1.09	1.13	1.03	1.19	1.07	1.09	1.07	1.16	1.00

Note

AAD, average absolute deviation; *B*_f,_ bias factor; *A*_f,_ accuracy factor; *b*_0_, *b*_1_, *b*_2_, *b*_3_, *b*_11_, *b*_22_, *b*_33_, *b*_12_, *b*_13_, *b*_23_ are the equation regression coefficients for intercept, linear, quadratic, and interaction coefficient, respectively. *R* ^2^ coefficient of determination.

**Table 9:** Coefficient of regression and R^2^, AAD, B_f,_ A_f_ for the mathematical models of the responses (textural properties)

Coefficient	Hardness	Deflection @peak	Energy to peak	Adhesiveness	Chewiness	Cohesiveness	Fracturability	Gumminess	Springiness	Stringiness	Force @break	Energy to break
*b*_0_	13.4560	4.7540	0.30	2.8160	5.6620	0.610	12.6780	8.0820	0.712	3.7800	−0.368	0.0360
*b*_1_	3.3112	−0.3863	0.00	3.2375	−0.4475	−0.0775	0.2675	0.21750	−0.11625	0.37250	−0.37375	−0.06250
*b*_2_	6.0500	−0.4175	0.0125	1.0975	0.3725	−0.0800	3.6425	1.81375	−0.1650	0.27875	−0.13750	0.09000
*b*_3_	5.5313	−0.5588	0.0075	3.3000	−0.7900	−0.1400	3.2525	0.27625	−0.20125	0.38875	−0.38375	0.01500
*b*_11_	−1.3218	−0.1120	−0.000	2.8657	−1.1197	−0.0100	−3.5903	−1.33475	−0.00100	−0.18750	−0.33225	0.06950
*b*_22_	−0.6592	−0.1545	−0.000	−2.5293	−0.5997	−0.02000	−1.1753	−0.59225	0.00150	−0.0600	0.29025	0.06950
*b*_33_	−1.8067	−0.4410	−0.0100	2.2108	−2.6248	−0.03000	−2.6002	−3.25725	−0.07600	0.0300	−0.25725	−0.07550
*b*_12_	0.4400	−0.2375	−0.005	0.6475	−0.4475	−0.00500	−2.2925	−0.0700	−0.0475	0.12750	−0.09500	−0.14000
*b*_13_	5.3925	−0.5600	0.005	5.8575	−0.7875	−0.13000	1.9775	0.0500	−0.155	0.33250	−0.69250	0.01500
*b*_23_	6.3700	−0.5725	0.0100	0.6225	−0.3325	−0.12000	4.9475	1.02750	−0.2175	0.3550	−0.06000	0.01500
*R* ^2^ (%)	89.37	95.66	75.16	74.59	75.92	94.07	80.04	72.71	93.73	95.43	73.41	72.03
AAD	0.19	0.03	0.20	0.15	0.14	0.07	0.16	0.21	0.15	0.02	0.18	0.21
*B*_f_	1.02	1.00	1.03	1.01	1.03	1.00	1.01	1.02	1.03	1.00	1.03	1.01
*A*_f_	1.17	1.03	1.18	1.13	1.20	1.07	1.21	1.20	1.13	1.02	1.01	1.02

Note

AAD, average absolute deviation; *B*_f,_ bias factor; *A*_f,_ accuracy factor; *b*_0_, *b*_1_, *b*_2_, *b*_3_, *b*_11_, *b*_22_, *b*_33_, *b*_12_, *b*_13_, *b*_23_ are the equation regression coefficients for intercept, linear, quadratic, and interaction coefficient, respectively. *R* ^2^ coefficient of determination.

### 3.5 Sensory analysis

Consumer acceptability tests were conducted to evaluate their preferences in terms of aroma, color, taste, crispiness, and overall acceptability of fried sausage samples and to know the level of acceptance based on the magnitude of their responses (likes and dislikes). The panelists thus used the corresponding sensory scores for each of the attributes as shown in Table 10. Results showed that there were significant differences (*p* < .05) in all the samples investigated and the goat meat sausage sample prepared at cooking time of 45 min, frying temperature of 150°C, and at time of 9 min were generally the most preferred and accepted by the sensory panelists, while sample with cooking time of 30 min, frying temperature of 170°C, and time of 9 min was the least preferred.

**Table 10:** Sensory properties of fried goat meat sausage

Runs	*X*_1_ (mins)	*X*_2_ (^o^C)	*X*_3_ (mins)	Aroma	Color	Taste	Crispiness	Overall Acceptability
1	60	170	9	7.60^abc^	7.00^a‐d^	7.50^ab^	6.20^cd^	7.35^ab^
2	45	150	9	7.55^a‐d^	6.85^bcd^	7.40^ab^	7.60^a^	7.55^a^
3	60	170	3	7.65^ab^	7.35^abc^	6.90^abc^	7.40^ab^	7.40^ab^
4	30	190	6	7.00^bcd^	6.95^a‐d^	7.25^abc^	5.65^d^	6.85^ab^
5	45	170	6	7.50^a‐d^	7.40^abc^	7.60^a^	6.90^abc^	7.50^a^
6	60	150	6	7.90^a^	7.55^ab^	7.35^ab^	6.35^bcd^	7.50^a^
7	45	190	3	6.75^cd^	6.60^cd^	6.70^abc^	5.40^d^	6.80^ab^
8	45	170	6	7.50^a‐d^	7.40^abc^	7.60^a^	6.90^abc^	7.50^a^
9	30	170	9	6.70^d^	7.80^a^	7.15^abc^	5.85^d^	6.60^b^
10	45	190	9	6.90^bcd^	7.30^abc^	6.60^abc^	7.15^abc^	6.75^ab^
11	30	170	3	7.30^a‐d^	7.15^a‐d^	7.30^ab^	5.80^d^	7.05^ab^
12	45	150	3	6.90^bcd^	6.95^a‐d^	6.50^bc^	5.45^d^	6.60^b^
13	45	170	6	7.50^a‐d^	7.40^abc^	7.60^a^	6.90^abc^	7.50^a^
14	45	170	6	7.50^a‐d^	7.40^abc^	7.60^a^	6.90^abc^	7.50^a^
15	45	170	6	7.50^a‐d^	7.40^abc^	7.60^a^	6.90^abc^	7.50^a^
16	60	190	6	7.25^a‐d^	6.40^d^	6.25^c^	7.30^ab^	7.25^ab^
17	30	150	6	7.10^a‐d^	7.55^ab^	6.80^abc^	7.35^ab^	7.10^ab^

Note

Values are means of duplicate determinations ± *SD*; Means value with different superscript within the same column are significantly different at *p* ≤ .05; *X*_1:_ (cooking time); *X*_2:_ (frying temperature); and *X*_3:_ (frying time).

## 4 CONCLUSION

The study showed that the frying kinetics obeyed the first‐order rate constant and the temperature dependency of moisture loss was higher compared to fat absorption of the fried goat meat sausage in all the samples. The optimization of the combined effects of the deep fat frying conditions was achieved using cooking time of 45 min, frying temperature of 150°C, and at time of 9 min with (*R* ^2^ > 0.9). These variables are therefore important viable alternative for the commercialization of quality goat meat sausages and other fried meat products in the food industry, and to produce aesthetically acceptable, shelf stable, and nutritionally fit products. However, further studies could be carried out on the storability or microbiological of the deep fat fried goat meat sausages.

## CONFLICT OF INTEREST

The authors declare that we do not have any conflict of interest.

## ETHICAL APPROVAL

This study was approved by the institutional ethical committee.

## INFORMED CONSENT

Written informed consent was obtained from all study participants.
